# Clinical significance of gastroesophageal reflux disease with minimal change: a multicenter prospective observational study

**DOI:** 10.1038/s41598-022-19408-w

**Published:** 2022-09-03

**Authors:** Noriaki Manabe, Takashi Joh, Kazuhide Higuchi, Katsuhiko Iwakiri, Takeshi Kamiya, Ken Haruma, Koji Nakada

**Affiliations:** 1 Division of Endoscopy and Ultrasonography, Department of Clinical Pathology and Laboratory Medicine, Kawasaki Medical School, 2-6-1 Nakasange, Kita-ku, Okayama, 700-8505 Japan; 2 Gamagori City Hospital, 1-1 Mukaida, Hirata-cho, Gamagori City, Aichi 443-8501 Japan; 3 Second Department of Internal Medicine, Osaka Medical and Pharmaceutical University, 2-7 Daigaku-machi, Takatsuki, Osaka 569-8686 Japan; 4 Department of Gastroenterology, Nippon Medical School Graduate School of Medicine, 1-1-5, Sendagi, Bunkyo-ku, Tokyo 133-8603 Japan; 5 Department of Medical Innovation, Nagoya City University Graduate School Medical Sciences, 1, Kawasumi, Mizuho-cho, Mizuho-ku, Nagoya 467-8601 Japan; 6 Department of General Internal Medicine 2, Kawasaki Medical School, 2-6-1 Nakasange, Kita-ku, Okayama 700-8505 Japan; 7 Department of Laboratory Medicine, The Jikei University Daisan Hospital, 4-11-1, Izumihon-cho, Komae City, Tokyo 201-8601 Japan

**Keywords:** Gastroenterology, Gastrointestinal diseases

## Abstract

Non-erosive reflux disease (NERD) is classified into grade N (no minimal change) and grade M (minimal change) based on the Los Angeles classification. However, few reports have described the clinical characteristics of grade M. This study was performed to clarify the clinical characteristics of grade M. Among 290 consecutive patients with gastroesophageal reflux disease (GERD), 45 patients with grade M, 62 patients with grade N, and 94 patients with grade A were compared with respect to clinical differences. The degree of symptom improvement after 4 weeks of proton pump inhibitor administration was also prospectively compared among the three groups. Grades N and M showed no or little difference in the patients’ backgrounds (including sex and body mass index), GERD/functional dyspepsia symptom scores, life dissatisfaction (diet, sleep, work, and mood), Short Form-8 (mental component summary) scores, and symptom improvement. In contrast, significant differences were present between grades M and A as well as between grades N and A. The overall results of our study suggest that the distinction between grade M and grade N is of little clinical significance from the viewpoint of clinical characteristics.

## Introduction

In recent years, changes in the structure of upper gastrointestinal (GI) tract diseases have occurred in Asian countries secondary to the Westernization of dietary habits, including changes in the gastric environment as represented by the decrease in the rate of *Helicobacter pylori* infection^1^. Among the various upper GI diseases, gastroesophageal reflux disease (GERD) is considered one of the most prevalent^2^. With these epidemiological changes, both accurate diagnosis of GERD and an appropriate treatment strategy are required. Recent reports from Western countries have indicated that clinicians tend to excessively diagnose GERD among patients complaining of gastric pain and/or heartburn and that proton pump inhibitors (PPIs) are being prescribed in excess of what is needed^3,4^.

GERD is classified into two types according to endoscopic findings: non-erosive GERD (NERD) and erosive GERD (ERD). The Los Angeles (LA) classification is widely used internationally for endoscopic classification of ERD^5^. In Japan, LA classification grade M (minimal change) has been introduced^6^. Several studies have investigated the differences among LA grades M, N, and A in terms of histology, acid exposure time, and esophageal motility dysfunction^7–11^. However, the clinical significance of classification of NERD into LA grades N and M remains unknown. To clarify the clinical characteristics of LA grade M, we compared the patients’ backgrounds, intensity of GERD and functional dyspepsia (FD) symptoms, impact on daily life, psychiatric bias, quality of life, and response to PPI treatment among LA grades M, N, and A.

## Results

### Patient characteristics

Among 290 consecutive patients with GERD, 45 (22.4%) patients with LA grade M, 62 (30.8%) patients with LA grade N, and 94 (46.8%) patients with LA grade A were enrolled at baseline. Of these patients, 108 (53.7%) were men and 93 (46.3%) were women. Their mean age was 57.1 ± 14.4 years, and their mean body mass index (BMI) was 23.6 ± 3.5 kg/m^2^. At baseline, the mean GERD symptom subscale (GERD-SS) score was 3.4 ± 1.2. The mean scores on the dissatisfaction with daily life scale (DS-SS) [i.e., dissatisfaction with eating (Q6), dissatisfaction with sleep (Q7), dissatisfaction with daily activities (Q8), and dissatisfaction with mood (Q9)] were 2.1 ± 1.1, 2.1 ± 1.1, 2.1 ± 1.0, and 2.7 ± 1.1, respectively. The mean Hospital Anxiety and Depression Scale (HADS) scores were 6.5 ± 3.5 and 5.6 ± 3.6, respectively. The mean acute (1-week recall) version of the health-related quality of life survey [Short Form-8 (SF-8)] physical component summary score was 45.4 ± 6.6, and the mean SF-8 mental component summary score was 46.3 ± 6.8.

### Comparison of patients’ backgrounds and clinical characteristics among the three groups

Table 1 compares the patients’ backgrounds and clinical characteristics among the three groups. For all items of the patients’ backgrounds and clinical characteristics, there were no significant differences between LA grades N and M. In contrast, there were significant differences in the BMI and DS-SS scores (sleeping, mood, and daily life) and differences with a tendency toward statistical significance in the DS-SS scores (eating and social activity) between patients with LA grades M and A. There were significant differences in the BMI and pretreatment FD-SS, FD-postprandial distress syndrome-SS, and DS-SS scores (eating, sleeping, social activity, mood and daily life) and differences with a tendency toward statistical significance in the GERD-SS scores between patients with LA grades M and A.

**Table 1:** Comparison of patients’ backgrounds and clinical characteristics among the three groups.

	Grade N (n = 62)		Grade M (n = 45)		Grade A (n = 94)		ANOVA	Tukey				
	Mean	SD	Mean	SD	Mean	SD	*p*-value	N vs. M	M vs. A	Cohen's *d*	N vs. A	Cohen's *d*
								*p*-value	*p*-value		*p*-value	
Age	57.0	13.6	56.7	16.8	57.4	13.8	0.970	0.993	0.969	0.04	0.990	0.02
**Sex**												
Men	25	40%	18	40%	65	69%	< 0.001					
Women	37	60%	27	60%	29	31%						
BMI	22.6	4.2	22.8	2.9	24.6	3.1	< 0.001	0.979	0.008	0.63	0.001	0.57
GERD-SS	3.6	1.3	3.6	1.0	3.2	1.3	0.072	0.985	0.214		0.097	0.33
FD-SS	3.2	1.1	3.0	1.0	2.7	1.1	0.006	0.594	0.181		0.005	0.51
FD-EPS	3.4	1.4	3.2	1.2	2.9	1.4	0.140	0.885	0.452		0.138	
FD-PDS-SS	3.1	1.2	2.8	1.1	2.4	1.1	0.001	0.472	0.101		0.001	0.6
Q6. eating	2.4	1.1	2.2	1.1	1.8	0.9	0.001	0.603	0.053	0.45	0.001	0.64
Q7. sleeping	2.3	1.1	2.4	1.2	1.9	1.0	0.012	0.919	0.029	0.47	0.048	0.41
Q8. social activity	2.4	1.0	2.2	1.0	1.8	1.0	0.003	0.789	0.072	0.42	0.004	0.53
Q9. mood	3.0	1.1	3.0	0.9	2.4	1.0	< 0.001	0.969	0.002	0.65	0.000	0.67
DS-SS	2.5	0.9	2.4	0.8	1.9	0.8	< 0.001	0.786	0.007	0.59	< 0.001	0.7
Anxiety score	6.9	3.6	6.7	3.2	6.1	3.5	0.307	0.959	0.567		0.318	
Depression score	6.1	3.9	5.7	3.8	5.1	3.4	0.246	0.864	0.620		0.230	
SF-8 PCS	44.6	6.5	44.9	6.3	46.0	6.8	0.384	0.968	0.636		0.230	
SF-8 MCS	45.1	6.6	44.9	6.6	47.8	6.7	0.018	0.993	0.057	0.43	0.230	

ANOVA, analysis of variance; SD, standard deviation; BMI, body mass index; GERD-SS, gastroesophageal reflux disease symptom subscale; FD-SS, functional dyspepsia symptom subscale; FD-EPS-SS, functional dyspepsia/epigastric pain symptom subscale; FD-PDS-SS, functional dyspepsia/postprandial distress symptom subscale; DS-SS, dissatisfaction with daily life subscale; SF-8, Short Form-8; PCS, physical component summary; MCS, mental component summary.

### Degree of symptom improvement after 4 weeks of PPI administration

**Figure 1:**
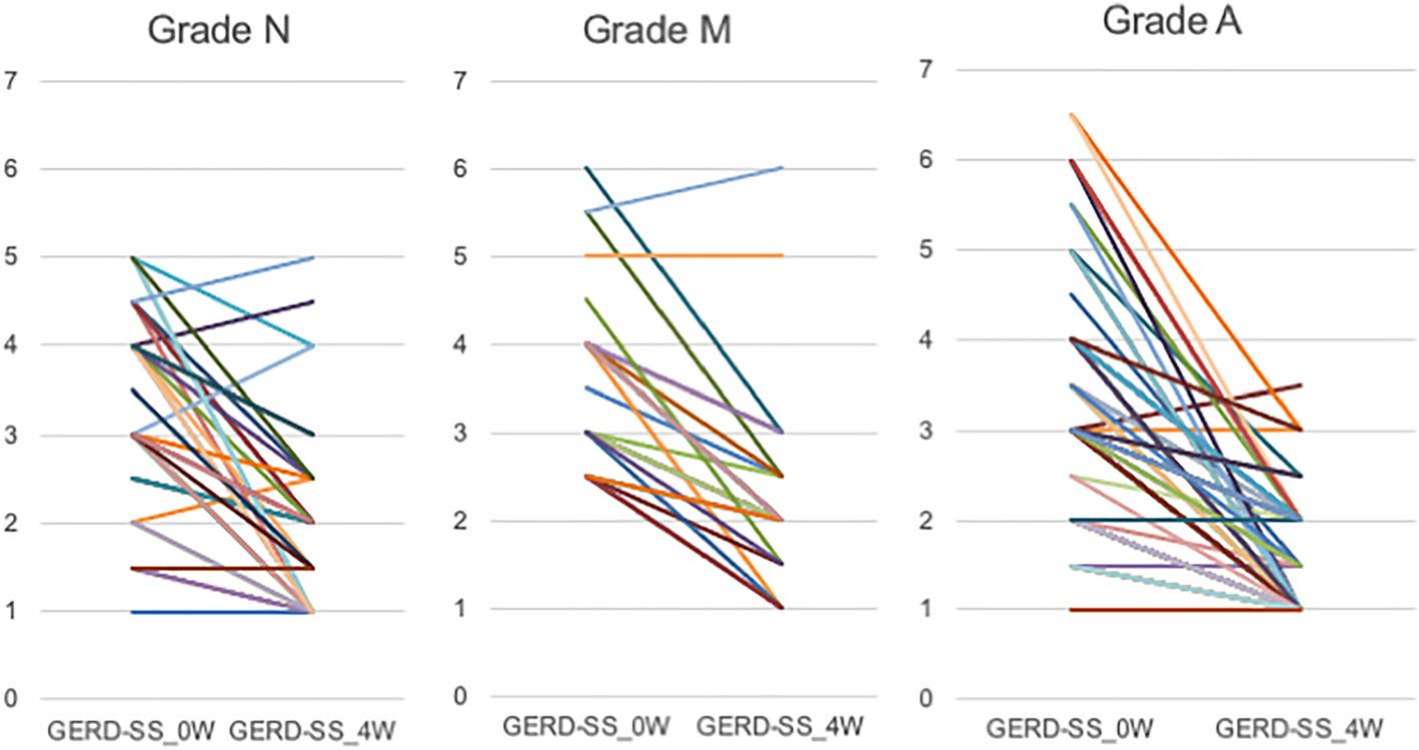
Changes in gastroesophageal reflux disease symptom subscale scores before and after 4 weeks of proton pump inhibitor administration.

**Figure 2:**
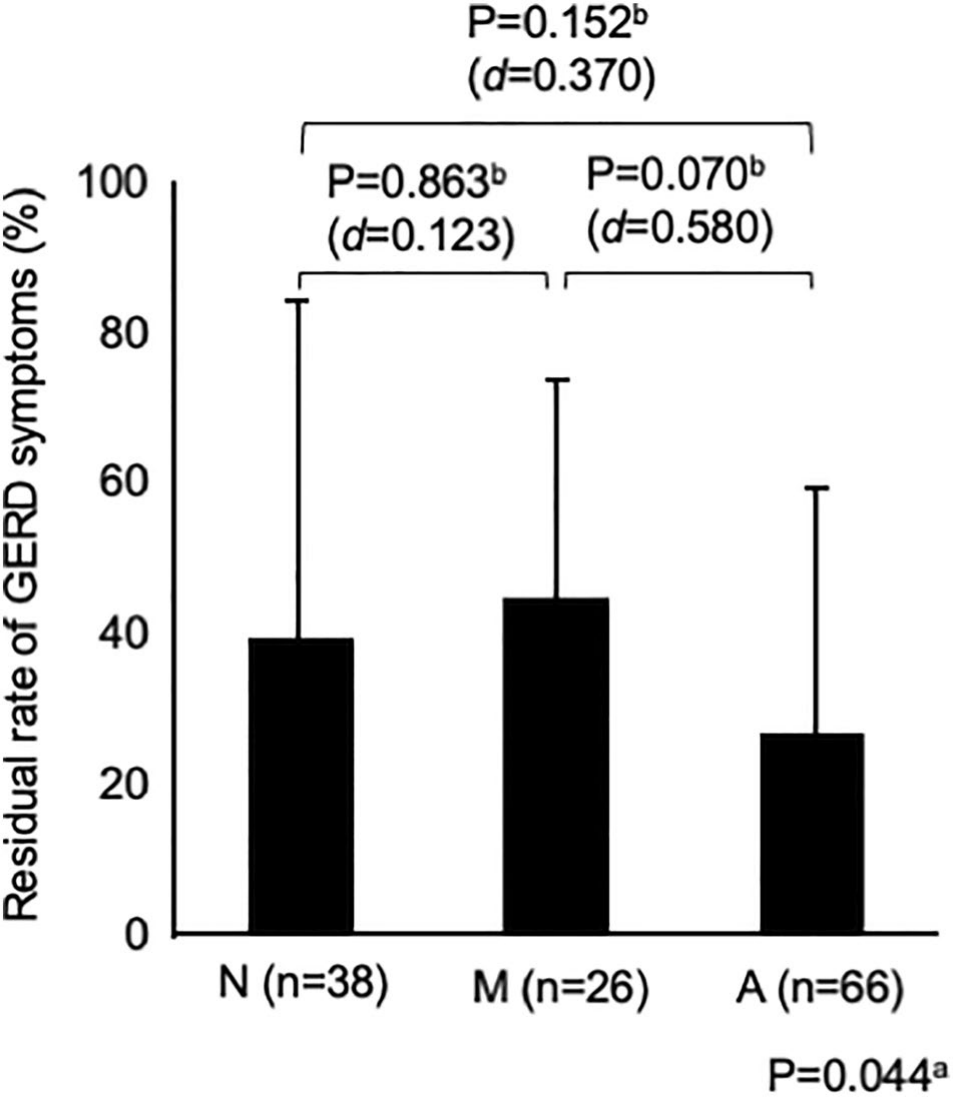
Comparison of symptom improvement among the three groups after 4 weeks of proton pump inhibitor administration: Residual rate of gastroesophageal reflux disease symptoms. NOTE: a = analysis of variance, b = Tukey’s honestly significant difference test, d = Cohen’s *d.*

**Figure 3:**
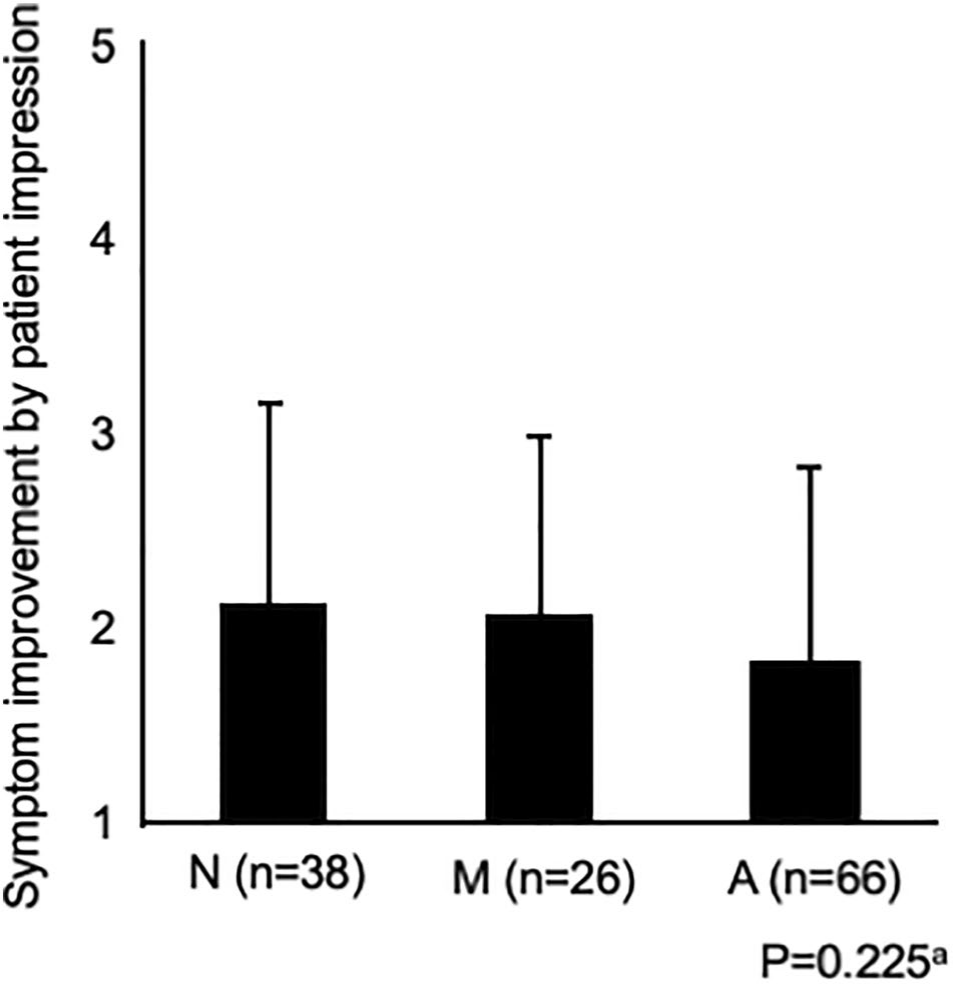
Comparison of symptom improvement among the three groups after 4 weeks of proton pump inhibitor administration: Symptom improvement through patient impressions. NOTE: a = analysis of variance.

**Figure 4:**
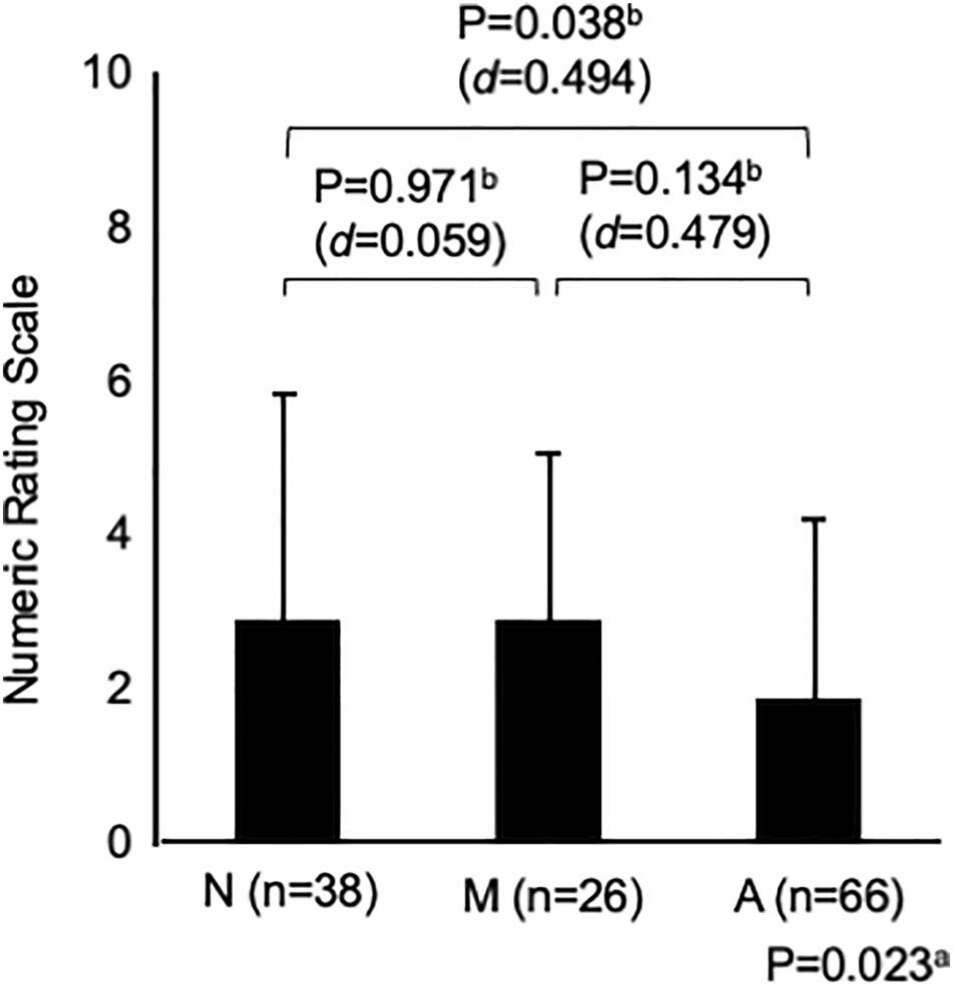
Comparison of symptom improvement among the three groups after 4 weeks of proton pump inhibitor administration: Global assessment scale. NOTE: a = analysis of variance, b = Tukey’s honestly significant difference test, d = Cohen’s *d.*

Figure 1 shows the changes in the GERD-SS scores before and after 4 weeks of PPI administration according to LA grades N, M, and A. Figure 2 shows the difference in the residual rate of GERD symptoms among the three groups. There was no significant difference in the residual rate of GERD symptoms among the three groups, although the residual rate tended to be lower in patients with LA grade A (Fig. 2). Figure 3 shows that there was no difference in symptom improvement by patient impressions among the three groups. Figure 4 shows the difference in the numeric rating scale score among the three groups. There was a significant difference between LA grades A and N, but no significant difference between LA grades N and M.

## Discussion

In this multicenter prospective observational study, the clinical characteristics of patients with LA grade M were similar to those of patients with LA grade N both before and during treatment; however, they were clearly different from those of patients with LA grade A. These findings indicate that it is clinically meaningful to distinguish patients with NERD from those with ERD, but it is not meaningful to distinguish between patients with and without minimal change.

Because upper GI endoscopy appears to be the only diagnostic modality that can diagnose ERD with high sensitivity and can also diagnose Barrett’s esophagus, endoscopy should be the first-line procedure when testing for ERD is necessary^12^. Most patients with GERD do not have endoscopic evidence of mucosal breaks; therefore, negative endoscopy findings do not exclude the diagnosis of GERD. In other words, endoscopic examination seems to be less sensitive for symptomatic GERD. Traditionally, a diagnosis of NERD has been given to patients with heartburn or acid regurgitation without any esophageal mucosal breaks; generally, patients with NERD tend to have negative pH tests^13^. The characteristics of acid reflux and the pattern of symptoms suggest that this patient population is heterogeneous. In Japan, LA classification grade M has been introduced to classify such patients more clearly^14^. However, this terminology has caused some confusion among endoscopists; research has shown that interobserver agreement on the endoscopic diagnosis of LA grade M is too low to be of clinical significance^15^. According to one review, at least two-thirds of patients with NERD have microscopic esophageal mucosal damage, such as dilated intercellular spaces, and this microscopic esophageal injury is considered a clinical sign of a response to PPI therapy^16^. Although minimal change such as redness of the esophageal mucosa can occur due to gastric acid reflux, this phenomenon can also occur even within the normal range of gastric acid reflux; therefore, the presence of minimal change does not mean that a patient necessarily has GERD. When the diagnosis of LA grade M is made by endoscopy, the condition is not the same as LA grade A but is the same as LA grade N^9,13^. This is consistent with our study results.

Several studies have focused on the pathogenesis of minimal change esophagitis^7,9^, but the results have been conflicting. Lei et al.^9^ studied the pathogenesis of minimal change esophagitis in 100 patients (esophagitis without minimal change, n = 52; esophagitis with minimal change, n = 48). The rate of effective peristalsis was comparable in patients with and without minimal change esophagitis (*p* = not significant). There was no difference in the esophageal acid reflux status or DeMeester score between the two groups (*p* = not significant). Additionally, intragastric acidity (pH < 4) was comparable in patients with and without minimal change esophagitis. The authors concluded that among patients with NERD, the disease characteristics in terms of esophageal acid exposure and motor dysfunction may be similar between patients with and without minimal change esophagitis^9^. This finding is consistent with our results. Another study from Japan examined the difference in the pathogenesis with respect to esophageal acid reflux using 24-h pH monitoring between patients with LA grade M and those with LA grade N^7^. There was no significant difference in the quality of life score or patients’ backgrounds between the two groups. However, 57.1% (8/14) of patients with grade M had a pH of < 4 for ≥ 4% of the total time (abnormal acid reflux), compared with only 11.8% (2/17) of patients with grade N (*p* = 0.018). Nevertheless, the median percent time with a pH of < 4.0 was 1.5% (range 0.0–11.1%) and 6.4% (range, 0.3%–14.9%) for grade N and grade M, respectively, which are not high. Therefore, it is possible that this difference was not large enough to result in a difference in PPI responsiveness.

This study has several limitations. First, the interobserver agreement on the endoscopic diagnosis of LA grade M is known to be too low. We did not examine the interobserver agreement among the endoscopists at all institutions with regard to an endoscopic diagnosis of minimal change. Before the study, we had an opportunity to present representative endoscopic images and explain the endoscopic diagnosis of minimal change, and we obtained a consensus from the endoscopists participating in the study. However, we do believe that the accuracy of the endoscopic diagnosis of GERD is reliable because all doctors involved in this study were actively engaged in the treatment of GERD on a daily basis and were endoscopists certified by the Japanese Society of Endoscopy. Second, 24-h pH-impedance monitoring and high-resolution esophageal manometry were not performed in this study; therefore, esophageal hypersensitivity, functional heartburn, and esophageal dysmotility may have been included in the NERD group. Third, because the LA grade was determined based on the endoscopy findings of patients who presented with symptoms that met the Montreal criteria, the NERD group may have included patients who previously had ERD and subsequently changed to NERD because of treatment or the natural history of the disease. Fourth, the power of the study was insufficient because of the small number of cases. Even when the effect size (Cohen’s *d*) was moderate (around 0.5), no significant difference was found between some patients, indicating that the study may not have sufficiently demonstrated a clinically meaningful difference. A study with a larger number of cases is desirable.

In conclusion, minimal change as an endoscopic classification of GERD is unnecessary in clinical practice. From the viewpoint of clinical characteristics, the classification of NERD should be sufficient for endoscopy of patients who have heartburn without mucosal breaks.

## Methods

### Study design

This multicenter prospective observational study was conducted at 29 institutions in Japan. One or more investigators per institution was a member of the GERD Society, a Japanese collaborative research group consisting of experts in clinical practice of GERD treatment. This study was conducted in accordance with the Declaration of Helsinki (sixth revision, 2008) after obtaining approval from the ethics committee of each institution or the central ethics committee of Nishi Clinic, Osaka, Japan. The study was registered with the University Hospital Medical Information Network Center Clinical Trials Registry in Japan (reference number UMIN000006614).

**Figure 5:**
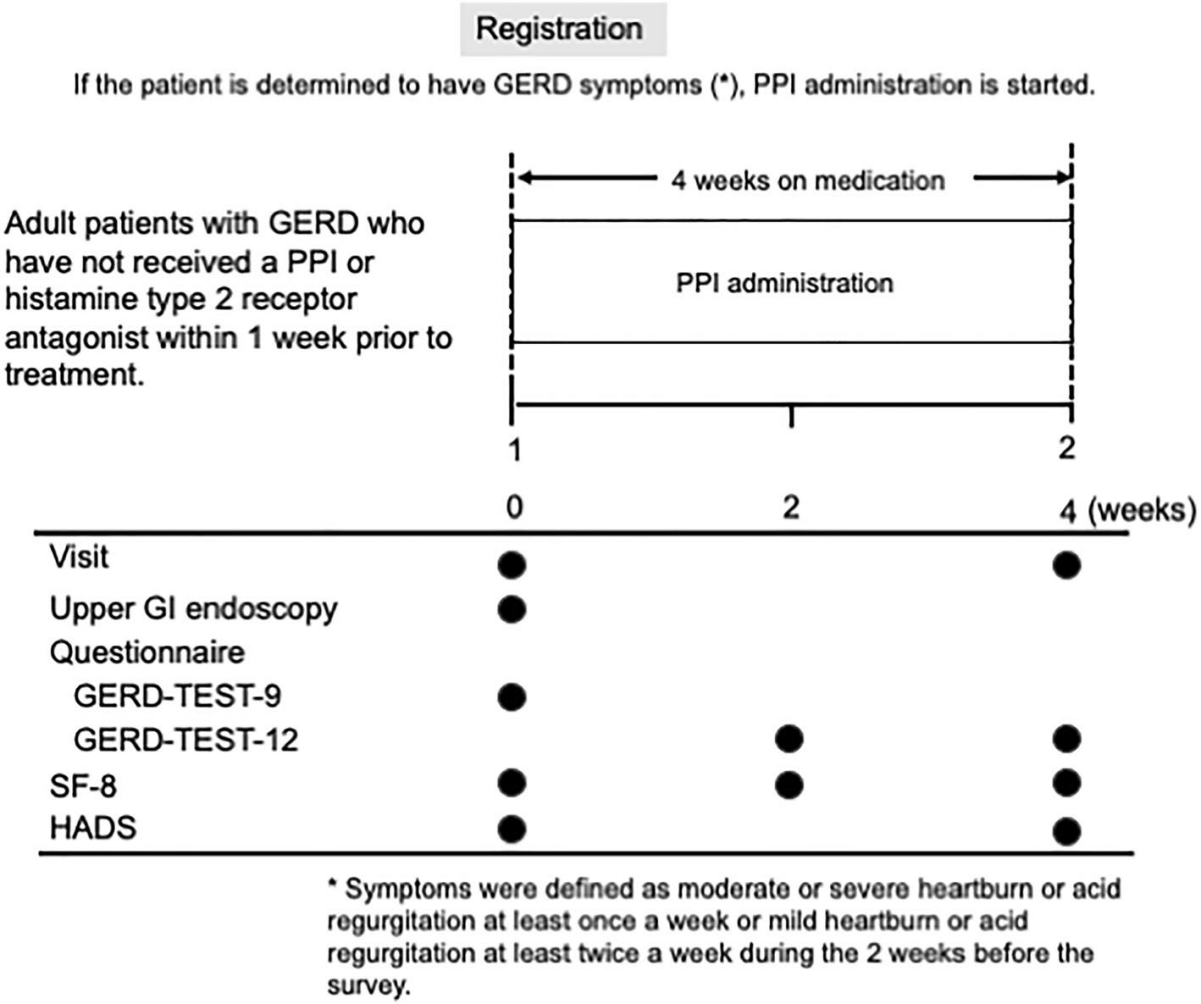
Study design.

The study design is shown in Fig. 5. Eligible patients were asked to complete the following questionnaires to evaluate patients’ clinical characteristics. Symptoms of GERD/FD and quality of life were assessed using the GERD-TEST^17^ and the SF-8^18^ at weeks 0, 2, and 4. Psychiatric assessments were conducted using the HADS^19^ at weeks 0 and 4. All questionnaires were completed by the study participants themselves and mailed to the data center.

### Patients

Outpatients with gastroesophageal reflux symptoms were enrolled in this study. Patients were considered to have gastroesophageal reflux symptoms if they had experienced moderate or severe heartburn or acid regurgitation at least once a week or mild heartburn or acid regurgitation at least twice a week during the 2 weeks prior to this study, according to the Montreal definition^20^. After upper GI endoscopy, the patients were administered a PPI at the dose approved in Japan; i.e., omeprazole at 20 mg once daily, lansoprazole at 30 mg once daily, or rabeprazole at 10 or 20 mg once daily.

**Figure 6:**
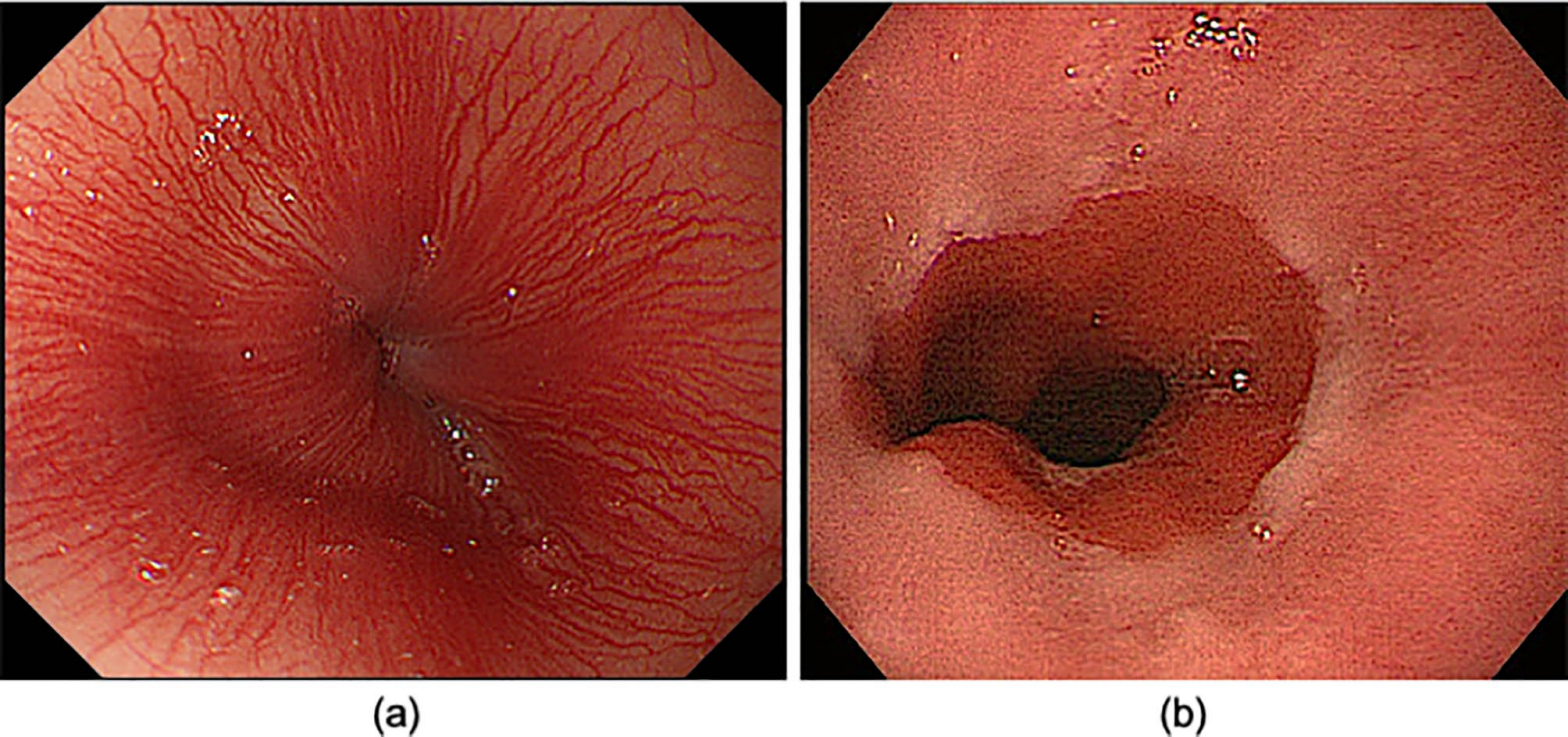
Typical endoscopic images of LA grades N and M. (**a**) LA grade N = no endoscopic changes in esophageal mucosa. (**b**) LA grade M = endoscopic appearance of discoloration of the esophageal mucosa.

The eligibility criteria were (1) an endoscopic diagnosis of LA grade N, M, or A GERD according to the modified LA classification system (Fig. 6)^6^; (2) age of > 20 years at the time of providing consent; and (3) provision of written informed consent.

The exclusion criteria were (1) concomitant or prior diseases that may affect the study results (e.g., Zollinger–Ellison syndrome, inflammatory bowel disease, irritable bowel syndrome, esophageal stricture, eosinophilic esophagitis, esophageal achalasia, malabsorption, cerebrovascular disease); (2) vomiting associated with other diseases, peptic ulcers except in the scar stage, or other symptoms of severe liver disease, renal disease, cardiac disease, psychiatric disease, metabolic disorder, neurological disease, or collagen disease; (3) confirmed or suspected malignancy; (4) history of GI surgery or vagotomy; (5) history of hypersensitivity to PPIs or their excipients; (6) eradication of *H. pylori* within 6 months prior to enrollment; (7) pregnancy, possible pregnancy, or lactation; (8) medication with a PPI or histamine type 2 receptor antagonist within 1 week prior to enrollment; and (9) ineligibility for the study as determined by the physician. Prohibited medications were those that may affect the study results (PPIs, histamine type 2 receptor antagonists, prokinetic agents, gastric mucosal protectants, and anticholinergics other than the study medication) and those that may interact with any of the study medications.

### Details of questionnaires for data collection

The GERD-TEST is a patient-reported 13-item questionnaire developed to investigate the symptoms of GERD and dyspepsia, their impact on the patient’s daily life, and the patient’s impression of the treatment. The GERD-SS is defined as the mean of the heartburn (Q1) and acid regurgitation (Q2) scores, and the FD symptom subscale (FD-SS) is defined as the mean of epigastric pain/burning symptoms (Q3) and postprandial distress symptoms [postprandial fullness (Q4) and early satiation (Q5)]. The DS-SS is divided into dissatisfaction with eating (Q6), dissatisfaction with sleep (Q7), dissatisfaction with daily activity (Q8), and dissatisfaction with mood (Q9). Questions 10 to 13 focus on the effects of PPI treatment. The details of the GERD-TEST have been discussed in our previous report^21^.

The SF-8 is a questionnaire used to assess patients’ health status and consists of a physical component summary and a mental component summary^22^. Other details about the SF-8 have been previously reported^22^.

The HADS is a well-established measure of psychiatric bias with subscales for anxiety and depression, each comprising seven items^19^. A higher score indicates a higher level of anxiety or depression. The anxiety and depression scores were compared among the three groups in the present study.

### Therapeutic response to PPI therapy

The efficacy of PPI therapy in patients with GERD was evaluated using the following three indices, as we previously reported^21^: (1) the GERD-SS residual symptom rate: 100 (%) × (Week 4 GERD-SS score − 1)/(Week 0 GERD-SS score − 1), (2) the patient’s impression of the treatment: Q11 score on GERD-TEST (score of the impression that GERD symptoms have improved compared with those before the current medication; 1 = very much improved, 2 = improved, 3 = somewhat improved, 4 = no change, and 5 = worsened), and (3) the numeric rating scale score for GERD symptoms (Q12 of GERD-TEST): a numerical rating of the relative intensity of GERD symptoms (0 points = no symptoms, 10 points = same severity of symptoms as before treatment).

### Statistical analysis

Data from patients who underwent upper GI endoscopy; completed questionnaires within 4 weeks before starting treatment; provided information on sex, age, height, and body weight; and had a medication adherence rate of ≥ 75% were analyzed. Patients with gastroesophageal reflux symptoms were divided into three groups according to the endoscopic findings: LA grade N, LA grade M, and LA grade A. Data are expressed as mean ± standard deviation. The statistical methods used to compare patients’ characteristics and treatment effects among the three groups were analysis of variance followed by Tukey’s test and Fisher’s exact test. The size of the difference between groups was evaluated by the effect size (Cohen’s *d*). Cohen’s *d* values of ≥ 0.20, ≥ 0.50, and ≥ 0.80 were defined as small, medium, and large effects, respectively^23^. JMP12.0.1 software (SAS Institute Inc., Cary, NC, USA) was used for data analysis, and a *p* value of < 0.05 was considered to indicate clinical significance.

### Ethics approval and consent to participate and publish

All procedures were in accordance with the ethical standards of the responsible committee on human experimentation (institutional and national) and the Helsinki Declaration of 1964 and later versions. Informed consent or a substitute was obtained from all patients for their participation in the study.

## Data Availability

The datasets used and/or analyzed during the current study are available from the corresponding author (NM) on reasonable request.
